# Supplementary material for: Image-Guided Intraoperative Assessment of Surgical Margins in Oral Cavity Squamous Cell Cancer: A Diagnostic Test Accuracy Review
Source: Diagnostics (Basel). 2023 May 25;13(11):1846. doi: 10.3390/diagnostics13111846 (PMC10252470; doi:10.3390/diagnostics13111846)
Supplement: Supplementary file 1 [file diagnostics-13-01846-s001.zip › diagnostics-2352719-supplementary/Supplementary Table S1 (A.1).pdf]

| | Risk of bias | Concerns regarding applicability |
|---|---|---|
| **Patient selection** | • Patient sampling (open)<br>• Was a consecutive or random sample of patients enrolled ? Y/N<br>• Was a case-control design avoided? Y/N<br>• Did the study avoid inappropriate exclusions ? Y/N<br>• Were inclusion and exclusion criteria clearly stated? Y/N<br>• **Could the selection of patients have introduced bias**?(Low/ unclear/high risk) | • Patient characteristics and sampling (open)<br>• Was the sample size large enough to represent the general characteristics of the population? Y/N<br>• Was the median follow-up of patients long enough to ensure correct estimation of the outcomes? Y/N<br>• **Are there concerns that the included patients and settings do not match the review question**?( Low/ unclear/high risk) |
| **Index test** | • Where the index test results interpreted without knowledge of the results of the reference standard? (open)<br>• If a threshold was used, was it pre specified? Y/N<br>• Was the number of readers sufficient to avoid potential misclassification? Y/N<br>• Where the skills of the operators developed enough to guarantee average standards? Y/N<br>• **Was masking applied?** ( Low/ unclear/high risk) | **Are there concerns that the index test, its conduct, or interpretation differ from the review question?** ( Low/ unclear/high risk) |
| **Reference standard** | • results of the reference standard (open)<br>• If a threshold was used, was it pre specified? Y/N<br>• Was the number of readers sufficient to avoid potential misclassification? Y/N<br>• Were the reference standards likely to correctly classify the target condition? Y/N<br>• Where the reference standard results interpreted without knowledge of the results of the index tests? Y/N<br>• **Could the reference standard, its conduct, or its interpretation have introduced bias?** ( Low/ unclear/high risk) | **Are there concerns that the target condition as defined by the reference standard does not match the review question?** ( Low/ unclear/high risk) |
| **Flow and timing** | • Flow and timing (open)<br>• Was there an appropriate interval between index test and reference standard? Y/N<br>• Did all patients receive the same reference standard? Y/N<br>• Where all patients included in the analysis? Y/N<br>• **Could the patient flow have introduced bias?** Low/ unclear/high risk) | \ |
