# Supplementary material for: Image-Guided Intraoperative Assessment of Surgical Margins in Oral Cavity Squamous Cell Cancer: A Diagnostic Test Accuracy Review
Source: Diagnostics (Basel). 2023 May 25;13(11):1846. doi: 10.3390/diagnostics13111846 (PMC10252470; doi:10.3390/diagnostics13111846)
Supplement: Supplementary file 1 [file diagnostics-13-01846-s001.zip › diagnostics-2352719-supplementary/Supplementary Table S2 (A.2).pdf]

Characteristics of excluded studies

Au 2022

**Reason for exclusion**	Oral presentation – 29 patients in total; retrospective cohort study . T1-T3 tumors evaluated by 2 experienced head and neck neuroradiologists and 2 pathologists. Results: Eight out of the 11 patients (73%) with infiltrative TS had resection margin <5 mm; 1 at 4.5 mm, 2 at 4 mm, 4 at 3 mm, and 1 at <1 mm. Tumors with ill-defined TB on US were 8.7 times (95% CI, 1.40-53.8) more likely to not have perineural invasion, when compared with those with sharp TB.

Clayburgh 2016

**Reason for exclusion**	outcomes evaluated were not in line with the main question of the systematic review – *ie IOUS-assessment of cancer of the oropharynx (image-guided surgery)*

De Konig2020

**Reason for exclusion**	outcomes evaluated were not in line with the main question of the systematic review- *difference between ioIOUS measurement of DOI and TT compared to histology. Surgical margins were NOT evaluated*

Green 2020

**Reason for exclusion**	outcomes evaluated were not in line with the main question of the systematic review – *ie IOUS-assessment of surgical margins in cancer of the oropharynx (among which also retropharyngeal lymph nodes)*

Lee Dong 2022

**Reason for exclusion**	the measured outcome was not in line with the question formulated in the review – ie *evaluating the impact of resection margins on long-term prognosis, regardless on imaging guidance*

McMahon 2020

**Reason for exclusion**	the object of the study was not in line with the question formulated in the review- ie *evaluating the impact of imaging on surgical margins on OTSCC- where imaging was only preoperative and also including CT*

Noorlag 2020

**Reason for exclusion**	the characteristics of the paper do not meet the inclusion criteria set in the systematic review- both intraoral US and MRI were undertaken before (10 days on average) surgery

Noorlag 2022

**Reason for exclusion**	the paper is a narrative review

wang 2020

**Reason for exclusion**	the measured outcome was not in line with the question formulated in the review – ie *evaluating the difference between ex-vivo MR DOI measurement and pathological DOI. also DOI >5 and 10 mm were evaluated but no mention to the surgical margins.*

Wu-Chia 2018

**Reason for exclusion**	the paper is a narrative review
