# Supplementary material for: Image-Guided Intraoperative Assessment of Surgical Margins in Oral Cavity Squamous Cell Cancer: A Diagnostic Test Accuracy Review
Source: Diagnostics (Basel). 2023 May 25;13(11):1846. doi: 10.3390/diagnostics13111846 (PMC10252470; doi:10.3390/diagnostics13111846)
Supplement: Supplementary file 1 [file diagnostics-13-01846-s001.zip › diagnostics-2352719-supplementary/Supplementary Table S3 (A.3).pdf]

Study	was the sample size large enough to represent the characteristics of the general population	was the number of readers sufficient to avoid potential misclassification?
Adrianseens 2023	No	No
Bubul 2021	No	Unclear
De Konig 2022	Unclear	Unclear
De Koning 2021	No	Yes
Giannitto 2021	No	Yes
Heidkamp 2019	No	Yes
Nilsson 2022	Unclear	Unclear
Steens 2017 - handsearching	No	Yes
Tarabichi 2018	No	Unclear
Zhang 2022	Yes	Yes

Study	Was a case-control design avoided?	Could the selection of patients have introduced bias?	Were all patients included in the analysis?	Could the patient flow have introduced bias?
Adrianseens 2023	Yes	Low risk	No	Low risk
Bubul 2021	No	Unclear risk	Yes	Unclear risk
De Konig 2022	No	Low risk	No	Unclear risk
De Koning 2021	No	High risk	Yes	Low risk
Giannitto 2021	Yes	Unclear risk	Yes	Low risk
Heidkamp 2019	Yes	Low risk	Yes	Low risk
Nilsson 2022	No	Low risk	Yes	Low risk
Steens 2017 - hand	Yes	Unclear risk	No	Low risk
Tarabichi 2018	Yes	High risk	Yes	Low risk
Zhang 2022	Yes	Low risk	Yes	Low risk

Study	were the skills of the operators developed enough to guarantee average standards?	were inclusion and exclusion criteria clearly stated	was masking applied
Adrianseens 2023	Unclear	Yes	Unclear
Bubul 2021	Yes	Yes	Yes
De Konig 2022	Yes	Yes	Unclear
De Koning 2021	No	Yes	Unclear
Giannitto 2021	Yes	No	Yes
Heidkamp 2019	Yes	Yes	Yes
Nilsson 2022	Yes	Yes	Unclear
Steens 2017 - hand	Yes	No	No
Tarabichi 2018	Unclear	No	No
Zhang 2022	Yes	Yes	Yes
